# Plasma leptin and insulin-like growth factor I levels during acute exacerbations of chronic obstructive pulmonary disease

**DOI:** 10.1186/1471-2466-9-11

**Published:** 2009-04-05

**Authors:** Prokopis Kythreotis, Ageliki Kokkini, Stavrina Avgeropoulou, Argyro Hadjioannou, Efgenia Anastasakou, Antonis Rasidakis, Petros Bakakos

**Affiliations:** 1 Department of Respiratory and Critical Care Medicine, Sotiria Chest Hospital, Athens, Greece; 2 Department of Microbiology, Immunology Laboratory, Sotiria Chest Hospital, Athens, Greece

## Abstract

**Background:**

Recent studies have provided evidence for a link between leptin and tumor necrosis factor-alpha (TNF-α). Insulin-like growth factor I (IGF-I) mediates the metabolic effects of growth hormone (GH). The GH axis is believed to be suppressed in chronic obstructive pulmonary disease (COPD). The aim of this study is to find out whether acute exacerbations of COPD are followed by changes in plasma leptin and insulin-like growth factor I (IGF-I) levels and furthermore, whether these changes are related to systemic inflammation.

**Methods:**

We measured serum leptin, IGF-I, TNF-α, interleukin 1β (IL-1β), interleukin 6 (IL-6) and interleukin 8 (IL-8) levels in 52 COPD patients with acute exacerbation on admission to hospital (Day 1) and two weeks later (Day 15). 25 healthy age-matched subjects served as controls. COPD patients were also divided into two subgroups (29 with chronic bronchitis and 23 with emphysema). Serum leptin and IGF-I were measured by radioimmunoassay and TNF-α, IL-1β, IL-6 and IL-8 were measured by ELISA.

**Results:**

Serum leptin levels were significantly higher and serum IGF-I levels significantly lower in COPD patients on Day 1 than in healthy controls (p < 0.001). A positive correlation was observed between leptin and TNF-α on Day 1 (r = 0.620, p < 0.001). Emphysematous patients had significantly lower IGF-I levels compared to those with chronic bronchitis both on Day 1 and Day 15 (p = 0.003 and p < 0.001 respectively).

**Conclusion:**

Inappropriately increased circulating leptin levels along with decreased IGF-I levels occured during acute exacerbations of COPD. Compared to chronic bronchitis, patients with emphysema had lower circulating IGF-I levels both at the onset of the exacerbation and two weeks later.

## Background

Weight loss commonly occurs in patients with chronic obstructive pulmonary disease (COPD) [1]. In a subgroup of COPD patients, weight loss is suggested to follow a stepwise pattern related to acute disease exacerbations [2]. Recently, cytokine-mediated metabolic derangements have been considered to be among the candidates responsible for weight loss in COPD patients [3,4]. It has been shown that tumor necrosis factor-α (TNF-α) circulating levels are increased in weight-losing COPD patients [3,4]. COPD is characterized by a systemic inflammatory response that may be even more pronounced during an acute exacerbation of COPD [5].

Leptin, a protein synthesized by adipose tissue and encoded by the *ob gene *plays an important role in the energy balance and its circulating concentrations are proportional to the amount of fat mass (FM). Inappropriately increased leptin levels are thought to induce metabolic effects underlying anorexia and loss of body weight in chronic diseases including COPD [6,7]. Administration of endotoxin or cytokines such as TNF-α or IL-1 produced a prompt and dose-dependent increase in serum leptin levels in both experimental animals [8] and humans [9]. In stable patients with emphysema, leptin was found to be positively related to plasma soluble TNF-receptor 55 [5]. The observed link between inflammatory cytokines and leptin led to the hypothesis that adipose tissue gene expression is regulated by inflammatory cytokines, which in turn could induce anorexia in acute or chronic inflammation.

Growth hormone (GH) is secreted by the anterior pituitary and mediates its major metabolic effects through activation of somatomedins, predominantly insulin-like growth factor I (IGF-I) [10]. In COPD, little is known about circulating GH or IGF-I concentrations. Some authors found a decrease in GH or IGF-I, others an increase [11]. IGF-I levels tended to be lower in stable and hospitalized COPD patients due to an exacerbation [12]. IGF-I mRNA levels were decreased in muscle biopsies from hospitalized patients due to an acute exacerbation of COPD [13]. In animal studies, recombinant IGF-I has been shown to ameliorate the protein catabolism and promote anabolism observed under hypoxic conditions [14]. However, daily administration of rGH increased lean body mass but did not improve muscle strength in COPD patients [15].

In our study, we measured cytokine (TNF-α, IL-1β, IL-6 and IL-8), leptin and IGF-I levels at the onset of a COPD exacerbation as well as 15 days later. The aim of this study was to elucidate two questions. Firstly, whether COPD exacerbations are accompanied by changes in circulating levels of leptin and IGF-I. Secondly, whether plasma IGF-I and leptin levels are related to those of cytokines as a possible reflection of the enhanced inflammatory status observed during a COPD exacerbation.

## Methods

### Patients

The patient group consisted of subjects consecutively admitted to our department due to an infectious exacerbation of COPD. COPD was defined according to GOLD criteria [16]. Irreversible chronic airflow obstruction was confirmed, i.e., < 10% improvement in FEV1 expressed as percentage of predicted after inhalation of a β2-agonist. An infectious exacerbation of COPD was defined when at least two of the following three criteria were fulfilled: (a) recent increase in dyspnea (b) increased sputum volume and (c) sputum purulence, provided that one of the two criteria is purulent sputum [16].

All patients were current or ex-smokers. Patients with a medical history of comorbid diseases, such as diabetes mellitus, heart failure, lung cancer, collagen vascular disease or disturbances of thyroid function were excluded from the study. The final study group consisted of 52 patients (43 men, 9 women). The patients were treated with a standard protocol of medication consisting of nebulized short-acting β2-sympathicomimetics (salbutamol), inhaled anticholinergics (ipratropium bromide) and intravenously administered prednisolone. Prednisolone was given in the same dose (50 mg) for 7 days and then directly stopped, without tapering the dose. Antibiotic treatment was given to all COPD patients for 7 days. During hospitalization 38 of the 52 patients received supplemental oxygen therapy, according to measured arterial blood gases.

The control group consisted of 25 healthy age-matched adults (19 men, 6 women). They had no medical illnesses, had normal physical examinations, blood counts, chemistries and showed no symptoms or signs of infection at the time of the study.

All participants were informed in detail of the characteristics of the study and written consent was obtained. The study was approved by the ethics committee of our hospital.

### Body composition

Body height was determined to the nearest 0.5 cm with subjects standing barefoot. Body weight was assessed to the nearest 0.1 kg by using a digital weighing chair while subjects wore light clothing and no shoes. Body mass index (BMI) was calculated according to the equation BMI = Weight (kg)/Height^2 ^(cm^2^). Fat mass (FM) was measured with bioelectric impedance analysis with the portable device OMRON BF 302, (OMRON Healthcare Europe B.V, Hoofddorp). The basic principles of bioelectric impedance analysis were described by Lukaski and colleagues [17].

### Lung function

FEV1 and FVC were measured with standard spirometric techniques (SPIRO 232 MORGAN, Model 435 P.K MORGAN, Ltd. Gillingham, Kent, England). Arterial blood gases were obtained once in healthy individuals and twice in COPD patients (upon admission and after 15 days) with the subject in the sitting position and breathing ambient air. Lung function values were expressed as a percentage of predicted [18]. Arterial blood gases were analysed on a blood gas analyzer (Rapidlab 855, Bayer Healthcare LLC Diagnostics Division, NY 10591, USA).

### Assessment of emphysema

The distinction between COPD patients with predominant emphysema and those with chronic bronchitis was based mainly on high-resolution computed tomography (HRCT). HRCT is a sensitive technique for the evaluation of the presence and severity of emphysema. In patients with emphysema the densitometric parameters differ substantially from the corresponding values in patients with chronic bronchitis and healthy control subjects. Five thin-section CT scans were obtained in each patient: two scans of the upper, two scans of the lower lung zones at 3 and 6 cm above and below the carina and one scan at the carina. The severity and extent of emphysema of each scan was visually scored on a four-point scale independently by two observers according to the direct observational method of Sakai [19]. For each of the lung sections, the score for the severity of emphysema was multiplied by the score for the extent and the resultant scores were subsequently summed to give a total HRCT score. Visual scores ranged from 0 (no emphysema) to 120 (severe emphysema). Patients with a score < 30 were subtyped as chronic bronchitis and patients with a score ≥ 30 were subtyped as emphysema. According to this assessment the 52 patients with COPD were divided into 29 patients with chronic bronchitis and 23 patients with emphysema.

### Collection and analysis of laboratory and inflammatory parameters

Blood samples were collected in the morning, between 8.30 and 9.30 AM, when patients were in the fasting state for at least 10 h twice in COPD patients (on hospital admission-Day 1, and 15 days later- Day 15) and once in healthy individuals. For the measurement of TNF-α, IL-1β, IL-6, IL-8, leptin and IGF-I, blood was collected in plastic tubes containing ethylenediamine-tetraacetic acid (EDTA) and was centrifuged (1,000 × g for 10 min) within an hour. The supernatants were stored at -70°C until analysis. Cytokines (TNF-α, IL-1β, IL-6, IL-8) were measured with ELISA (BMS 223/3, BMS 224, BMS 213/2 and BMS 204, Bender MedSystems, MedSystems Diagnostics GmbH, Vienna, Austria Europe, respectively). Leptin and IGF-I were measured with radioimmunoassay (RIA, Active Human Leptin IRMA DSL – 23100, Diagnostic Systems Laboratories, Inc., USA and IGF-I 100 T KIT – Catalog No. 40-2100, Nichols Institute Diagnostics, USA, respectively). The lower detection limit of leptin was 0.10 ng/ml. The intra- and interassay variation coefficient were 4.9 and 6.6% respectively. The lower detection limit of IF-I was 0.06 ng/ml. The intra- and interassay coefficient were 3% and 9.8% respectively. All measured values for leptin and IGF-I were above the lower detection limit.

### Statistical analysis

Data were expressed as mean ± SD or as median (25–75 percentile) if they were not normally distributed. Statistical analysis was performed using nonparametric tests (Mann-Whitney U test for comparison between groups and Wilcoxon for comparison within groups). Dichotomized Δ was calculated by the formula Δ = D15-D1. Therefore ΔTNF-α = TNF-αD1-TNF-αD15, ΔLeptin = LeptinD1-LeptinD15 etc. The relations between continuous variables were evaluated with Spearman's rank correlation technique. Statistical significance was accepted at p < 0.05. Data were analysed using SPSS (Statistical Package for the Social Sciences, version 14.0 for Windows, SPSS Inc, Chicago, IL).

## Results

### Study group

The mean age of COPD patients was 65.8 ± 8.3 years and the control group 65.9 ± 9.6 years. Clinical characteristics of the COPD patients (on admission-Day 1) and the healthy controls are given in Table 1.

**Table 1:** Characteristics of the two groups

	**COPD patients (n = 52)** **43 M, 9 F** **(Mean ± SD)**	**Healthy subjects (n = 25)** **19 M, 6 F** **(Mean ± SD)**	**p**
**Age, yr**	65,8 ± 8,3	65.9 ± 9.6	0.789

**Weight, kg**	71,1 ± 13,6	77.2 ± 10.1	**0.037**

**Height, cm**	166,48 ± 7,11	167.56 ± 6.91	0.669

**BMI, kg/m^2^**	25,6 ± 4,1	27.9 ± 4.1	**0.029**

**Fat mass, kg**	21,5 ± 6,6	25.9 ± 7.3	**0.011**

**Fat free mass, kg**	49,6 ± 9,0	51,2 ± 7,9	0.427

**% FM**	29,8 ± 5,1	31.6 ± 6.3	0.211

**FVC %Pred**	62,4 ± 14,3	92.6 ± 6.6	**0.000**

**FEV_1_%Pred**	44,1 ± 11,4	89.9 ± 8.0	**0.000**

**PaO_2_mmHg**	58,2 ± 12,5	87.6 ± 4.6	**0.000**

**PaCO_2_mmHg**	47,1 ± 10,8	39.2 ± 2.5	**0.000**

Patients with COPD had significantly lower body weight, BMI and fat mass than did the control subjects. The COPD patients had severe airflow limitation, decreased PaO_2 _and increased PaCO_2_values. The control subjects had normal % FVC and % FEV1 on spirograms.

### Course of the parameters during the exacerbation

#### Inflammatory parameters

Compared to their concentrations in the healthy subjects, TNF-α, IL-1β, IL-6 and IL-8 were significantly higher in the patients with COPD on D1. By Day 15 cytokine levels did not differ from those of healthy controls with the exception of IL-8. Although IL-8 levels were decreased on D15 in COPD patients, they still significantly differed from healthy subjects (Table 2).

**Table 2:** Concentrations of plasma cytokines, leptin and IGF-I in healthy controls compared to patients with COPD exacerbation on admission to the hospital (D1) and after two weeks (D15)

**Markers** **Median (25–75 percentile)**	**COPD patients (D1)** **(n = 52)**	**Healthy subjects** **(n = 25)**	**COPD patients** **(D15)** **(n = 52)**
**TNF-α (pg/ml)**	63.9(46.7–84.5)* ††	14 (9.2–15.8)	14.3 (10.5–19.9)

**IL-1β (pg/ml)**	12.5 (8.8–16.9) * ††	3.6 (2.6–4)	3.8 (2.7–5.2)

**IL-6 (pg/ml)**	23 (17.7–44.6) * ††	9.7 (7.5–12.3)	10.6 (6.5–14.9)

**IL-8 (pg/ml)**	39.8 (29.2–56.3) * ††	13.7 (10–19)	18.5 (12.3–24) †

**Leptin (ng/ml)**	33.3 (16.7–45.5) * ††	9.6 (4.5–13.7)	15.7 (8.5–26) †

**IGF-1 (ng/ml)**	59.7 (44.3–76) * ††	133.2 (124–164.5)	96.6 (82.9–116.8) *

**Leptin/% FM**	1.1 (0.6–1.55) * ††	0.30 (0.16–0.43)	0.5 (0.26–0.94) *

Between healthy subjects and COPD patients * p < 0.01 † p < 0.05

Between COPD patients D1 and D15 †† p < 0.01

In COPD patients hospitalized due to an acute exacerbation, TNF-α, IL-1β, IL-6 and IL-8 levels on D1 were significantly higher compared to the levels measured on D15 (Table 2).

When COPD patients who received oxygen therapy were compared to those who did not, no difference in cytokine levels was detected.

#### Leptin

The levels of leptin in COPD patients with exacerbation were significantly higher on D1 and still on D15 compared to healthy subjects [33.3 (16.7–45.5)] ng/ml, p < 0.0001 and [15.7 (8.5–26)] ng/ml, p = 0.022 respectively, versus [9.6 (4.5–13.7)] ng/ml). Leptin concentration decreased significantly from D1 to D15 of the COPD exacerbation (p < 0.0001). After dividing leptin by %FM, the same pattern was seen. Thus, leptin/%FM was significantly elevated on D1 and nearly significantly increased on D15 compared to healthy subjects (p <0.001 and p = 0.008 respectively).

#### Insulin-like growth factor I (IGF-I)

Compared to its concentration in the healthy subjects [133.2 (124–164.5)] ng/ml), plasma IGF-I was significantly lower in COPD patients on D1 of the exacerbation and remained lower on D15 [59.7 (44.3–76)] ng/ml, p <0.001 and [96.6 (82.9–116.8)] ng/ml, p < 0.001 respectively versus healthy subjects). IGF-I levels were significantly increased from D1 to D15 throughout the exacerbation (p < 0.001).

#### Chronic bronchitis and emphysema

Characteristics of the study group stratified into the COPD subtypes (23 patients with emphysema and 29 patients with chronic bronchitis) are given in Table 3.

**Table 3:** Characteristics of patients with chronic bronchitis and emphysema

	**Chronic bronchitis** **(n = 29)** **25 M, 4 F**	**Emphysema** **(n = 23)** **18 M, 5 F**	**p**
**Age, yr**	68.9 ± 9.3	70.8 ± 7.0	0.524

**Weight, kg**	75.3 ± 14.1	65.9 ± 11.4	**0.022**

**Height, cm**	166.44 ± 7.10	166.52 ± 7.29	0.971

**BMI, kg/m^2^**	27.1 ± 4.2	23.7 ± 3.1	**0.003**

**% FM**	31.4 ± 4.5	27.9 ± 5.2	**0.047**

**Fat mass, kg**	23.7 ± 6.4	18.7 ± 5.7	**0.006**

**Fat free mass, kg**	51,5 ± 9,7	47,2 ± 7,7	0.058

**FVC %Pred**	65.7 ± 14.1	58.1 ± 13.8	**0.032**

**FEV_1_%Pred**	47.4 ± 11.2	39.2 ± 9.9	**0.005**

**PaO_2_mmHg**	59,4 ± 13,3	56,6 ± 11,6	0.256

**PaCO_2_mmHg**	49,6 ± 12,0	41,1 ± 9,5	0.276

Patients with emphysema were characterized by a significantly lower FVC and FEV1 compared to those with chronic bronchitis. Emphysematous patients had also a significantly lower BMI owing to a significantly lower FM (mean difference 5.04 kg). No difference was found in PaO_2 _and PaCO_2 _between patients with chronic bronchitis and emphysema.

**Figure 1:**
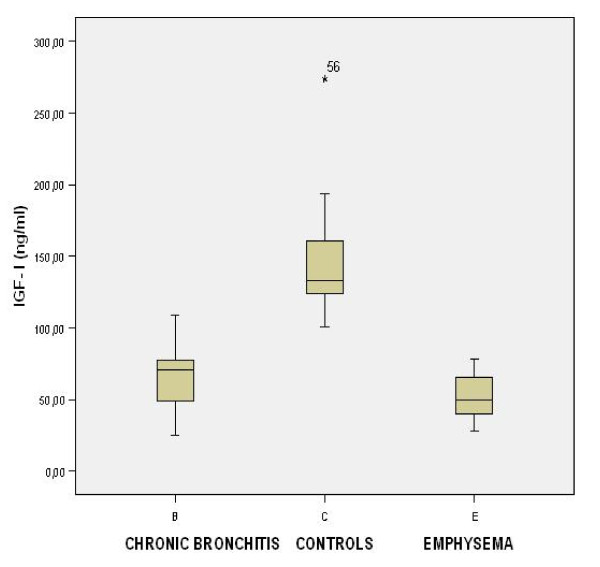
**IGF-I in chronic bronchitis (B), emphysema (E) and control group (C) on admission to the hospital (D1)**. Significant differences between B-E (p = 0.003), B-C (p = 0.000) and E-C (p = 0.000)

No differences were seen in the concentrations of TNF-α, IL-1β, IL-6, IL-8, leptin and leptin/% FM between the two groups of patients on D1 and D15. On the contrary, IGF-I was significantly lower in emphysematous patients compared to patients with chronic bronchitis both on D1 and D15 [49.5 (40.1–65.4)] versus [70.5 (48.6–78.5)] ng/ml, p = 0.003 on D1 and [84.7 (74.4–96.9)] versus [112.7 (92.4–124.1)] ng/ml, p < 0.001 on D15) (Table 4 and Table 5, Figure 1).

**Table 4:** Concentrations of plasma cytokines, leptin and IGF-I in patients with chronic bronchitis and emphysema on admission to the hospital (D1).

**Markers** **Median (25–75 percentile)**	**Chronic bronchitis** **(n = 29)**	**Emphysema** **(n = 23)**	**p**
**TNF-α (pg/ml)**	63.2 (45.5–85.7)	65 (47.2–80.2)	0.612

**IL-1β (pg/ml)**	12.6 (8.8–15.6)	12.3 (8.4–21.3)	0.941

**IL-6 (pg/ml)**	23.9 (19.7–40.4)	20.6 (15.9–52.6)	0.423

**IL-8 (pg/ml)**	39.7 (28.9–56.2)	42 (34.3–62.7)	0.574

**Leptin (ng/ml)**	37.7 (18.1–50.9)	32.5 (15.2–44.9)	0.214

**IGF-1 (ng/ml)**	70.5 (48.6–78.5)	49.5 (40.1–65.4)	**0.003**

**Leptin/% FM**	1.1 (0.62–1.61)	1.09 (0.6–1.55)	0.619

**Table 5:** Concentrations of plasma cytokines, leptin and IGF-I in patients with chronic bronchitis and emphysema on D15.

**Markers** **(Median (25–75 percentile)**	**Chronic bronchitis** **(n = 29)**	**Emphysema** **(n = 23)**	**p**
**TNF-α (pg/ml)**	13.7 (10.5–19.8)	15.1 (10.5–20.1)	0.632

**IL-1β (pg/ml)**	3.9 (3.4–5.8)	3.7 (2–4.7)	0.273

**IL-6 (pg/ml)**	10.6 (7.4–14.1)	10.4 (5.8–16.2)	0.706

**IL-8 (pg/ml)**	17.9 (11.8–23.2)	19 (12.3–26.6)	0.612

**Leptin (ng/ml)**	15.6 (7.9–28.6)	15.7 (8.4–22.8)	0.847

**IGF-1 (ng/ml)**	112.7 (92.4–124.1)	84.7 (74.4–96.9)	**0.000**

**Leptin/% FM**	0.45 (0.27–0.98)	0.56 (0.25–0.81)	0.768

#### Correlation analyses

A strong correlation between leptin and % FM was observed in controls (r = 0.780, p < 0.001).

In order to elucidate the possible relationship of circulating cytokine levels with plasma leptin and IGF-I concentrations correlation analysis was performed on D1 and D15.

**Figure 2:**
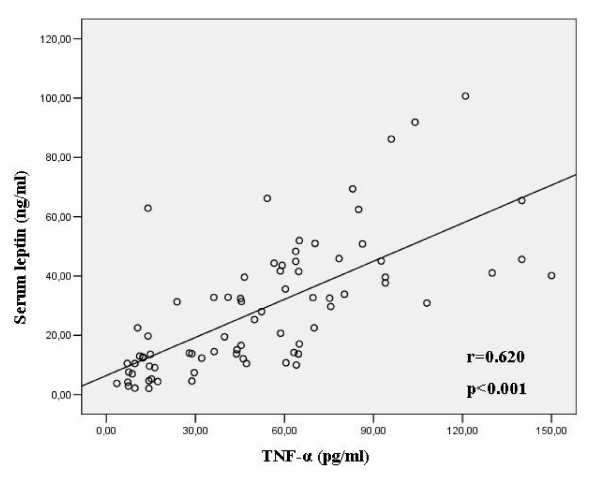
**Leptin is significantly related to TNF-α in COPD patients on Day 1 of the exacerbation**.

On D1 of the exacerbation, TNF-α demonstrated a significant positive correlation with leptin as well as with leptin adjusted to % FM (r = 0.620, p < 0.001 and r = 0.650, p < 0.001)(Figure 2). This correlation between TNF-α and leptin was true even when we studied patients with chronic bronchitis and emphysema separately (r = 0.719, p < 0.001 and r = 0.505, p = 0.014 respectively). TNF-α was also positively correlated with leptin/% FM in both chronic bronchitis and emphysema (r = 0.774, p < 0.001 and r = 0.499, p = 0.015 respectively). Moreover, the change in TNF-α levels (ΔTNF-α) was significantly related to the change in leptin levels (ΔLeptin) (r = 0.545, p < 0.001).

On D15, no significant correlations could be revealed between any of the parameters.

IGF-I was not found to be related to any of the inflammatory cytokines neither on D1 nor on D15.

## Discussion

The present study was performed to examine circulating levels of leptin and IGF-I at the onset of COPD exacerbations (D1) and two weeks later (D15) and furthermore to investigate the relationship of plasma leptin and IGF-I concentrations with cytokine concentrations as a possible reflection of the enhanced inflammatory status observed during the COPD exacerbation.

We found that COPD exacerbations are characterized by increased levels of leptin and the proinflammatory cytokines TNF-α, IL-1β, IL-6 and IL-8 and decreased levels of IGF-I on D1. We also found that two weeks after the onset of the exacerbation (D15) cytokine levels did not significantly differ from those of healthy subjects, with the exception of IL-8. IL-8 levels, although significantly decreased in COPD patients between D1 and D15, they still remained higher on D15 compared to healthy subjects. Although circulating plasma leptin decreases and plasma IGF-I increases, they still remained different compared to healthy controls on D15. Leptin was positively related to TNF-α on D1 of the exacerbation and this correlation also applied to both subgroups of COPD patients, thus chronic bronchitis and emphysema. Finally, we found that IGF-I levels were significantly lower to emphysematous patients compared to patients with chronic bronchitis both on D1 and on D15. Leptin and cytokine levels did not differ between patients with chronic bronchitis and emphysema.

The elevated levels of TNF-α, IL-1β, IL-6 and IL-8 on admission to the hospital (D1), observed in our study are in keeping with numerous studies that have reported increased levels of circulating cytokines in the peripheral circulation of patients with COPD [3,20,21]. These abnormalities, albeit seen in clinically stable COPD patients, they are generally more pronounced during exacerbations of the disease [21]. The systemic inflammatory response is ameliorated 15 days after the onset of the exacerbation, as shown by the decrease in cytokine levels.

Circulating leptin levels were high on D1 of the exacerbation and remained elevated on D15 in COPD patients compared to healthy subjects. The elevated leptin concentrations during COPD exacerbation are in keeping with the results of other studies [7] and likely represented an up-regulation of leptin mRNA resulting in an enhanced leptin production that might have been induced by several factors. The positive correlation between leptin and TNF-α which was seen in our COPD patients on D1 of the exacerbation, supports an inflammatory-related disturbance in leptin metabolism in COPD. This correlation was true even when we studied separately the two subgroups of patients (chronic bronchitis – emphysema). It has been shown that the administration of endotoxin or cytokines such as TNF-α and IL-1 can increase serum leptin levels both in hamsters and humans [8,9]. A positive correlation between leptin and soluble TNF-α receptors (sTNF-R55) was also found during COPD exacerbations in the study by Creutzberg et al [7]. In a recent study by Calikoglu et al serum leptin and TNF-α levels were increased in COPD patients with exacerbation in comparison to COPD patients with stable disease and healthy controls [22]. Moreover, and in accordance with our findings, a strong correlation between TNF-α and leptin was observed only in COPD patients with exacerbation but not in stable COPD patients and healthy individuals [22]. Takabatake et al recently reported that serum leptin levels were significantly lower in COPD patients than healthy controls [23]. However, this study included patients with stable disease who were free of symptoms for at least three months. The relationship between the change in TNF-α levels (ΔTNF-α) and the change in leptin levels (ΔLeptin) observed in our study, further supports a TNF-α-related disturbance in leptin during COPD exacerbations.

A reason for the high leptin concentrations might have been the glucocorticosteroid treatment and that presents a limitation of our study. Reports concerning the effects of corticosteroids on leptin are contradictory. Two days of oral dexamethasone (1.5 mg/day) in healthy subjects resulted in significantly increased serum concentrations of leptin [24]. In another study, administration of dexamethasone for 4 days (2.5 mg/day) to healthy subjects also induced a significant increase in plasma leptin concentrations [25]. Glucocorticosteroids may have a stimulating effect on leptin via the induction of insulin resistance, as glucose and insulin are also able to induce leptin expression [26]. However, Tataranni et al demonstrated that acute intravenous administration of glucocorticosteroids or prolonged oral treatment did not affect serum leptin levels [27]. Our COPD patients had severe airflow obstruction and it was necessary to introduce steroid treatment to improve their clinical status. However, samples on D1 were obtained before the first corticosteroid administration and on D15 7 days after the last intravenous administration of prednisolone, according to the protocol used. Regarding the effects of adrenergic stimulation on leptin metabolism, no studies have been performed with specific β2-adrenergic stimulants such as salbutamol. Intravenous infusion of isoprenaline in young healthy volunteers resulted in a maximal suppression of plasma leptin of 20% of baseline values after 2 h, but in the recovery period of 1 h, leptin concentrations rapidly returned to normal [28]. In our study COPD patients received salbutamol by nebulizer and not intravenously.

Limited data is available regarding circulating levels of IGF-I in COPD. Growth hormone (GH) mediates its major metabolic effects predominantly through IGF-I [10]. The GH axis is suppressed in chronic diseases and this may partly explain the low IGF-I levels. However, increased concentrations of GH have been found in COPD patients, especially in those with hypoxaemia [29].

In our study, patients with COPD exacerbation had significantly lower IGF-I levels on D1 and also on D15 compared to healthy controls. Our finding of low IGF-I levels is compatible with other studies. Casaburi et al have also reported low levels of IGF-I in COPD patients [30]. Spruit et al found that IGF-I levels tended to be lower in patients with COPD exacerbation than in healthy subjects [12].

We are aware that the bioavailability and the effects of IGF-I are influenced by IGF-I binding proteins (IGFBP). Therefore, an increase in circulating levels of these proteins may decrease the levels of free IGF-I [31]. Cytokines increase IGFBP-1 and IGFBP-4 and this results in a decreased free IGF-I fraction. IL-6 performs its suppressive activity on IGF-I via increased production of its binding protein [32].

It is interesting to note that IGF-I levels were lower in patients with emphysema compared to those with chronic bronchitis, both at the onset of the exacerbation and 15 days later, whereas cytokine and leptin levels did not differ between the two subgroups. The decline in IGF-I levels may itself be deleterious. IGF-I mRNA levels were decreased in muscle biopsies from hospitalized patients due to an acute exacerbation of COPD [13]. Moreover, it has been demonstrated that pulmonary rehabilitation leads to an increase in muscle mRNA expression of IGF-I in COPD patients [33]. COPD patients often present with hypoxia during exacerbations. In animal studies, recombinant IGF-I has been shown to ameliorate the protein catabolism observed under hypoxic conditions and to promote anabolism [14]. The decreased levels of this anabolic factor, especially in emphysematous patients further enhance its possible role in metabolic derangements of COPD and mainly emphysema. The lower body weight, usually observed in patients with emphysema may be related to the lower IGF-I levels found in these patients, particularly during exacerbations. Low levels of anabolic hormones could act synergistically with the catabolic activity of cytokines and leptin and play a role in the weight loss and decreased muscular mass usually seen in COPD patients and mainly those with emphysema. This may also have important therapeutic implications, since an increase in anabolic factors in COPD patients, particularly during exacerbations may have a protective metabolic effect.

## Conclusion

During an acute exacerbation of COPD, elevated levels of the proinflammatory cytokines TNF-α, IL-1β, IL-6 and IL-8 and increased levels of leptin and decreased levels of IGF-I are observed. It seems that, although systemic inflammation, in terms of cytokines, is restored relatively quickly, the coexistent disturbance of leptin and IGF-I is preserved for a longer period of time.

Lower levels of the anabolic factor IGF-I are observed in emphysema compared to chronic bronchitis and this may be related to the more pronounced metabolic derangements observed in this subgroup. Further studies are needed to elucidate the role of increased leptin and decreased IGF-I levels during COPD exacerbations and their possible relationship with energy imbalance observed in these patients.

## Competing interests

The authors declare that they have no competing interests.

## Authors' contributions

PK and PB conceived of the study, and participated in its design and coordination and drafted the manuscript. AK, AH and EA carried out the immunoassays. SA contributed to the acquisition of the data and the statistical analysis. AR participated in its design and drafted the manuscript. All authors read and approved the final manuscript.

## Pre-publication history

The pre-publication history for this paper can be accessed here:


